# Effectiveness of a WHO self-help psychological intervention to alleviate stress among healthcare workers in the context of COVID-19 in China: a randomised controlled trial

**DOI:** 10.1017/S2045796024000106

**Published:** 2024-03-07

**Authors:** Jinghua Li, Rui Luo, Pengyue Guo, Dexing Zhang, Phoenix K. H. Mo, Anise M. S. Wu, Meiqi Xin, Menglin Shang, Yuqi Cai, Xu Wang, Mingyu Chen, Yiling He, Luxin Zheng, Jinying Huang, Roman Dong Xu, Joseph T. F. Lau, Jing Gu, Brian J. Hall

**Affiliations:** 1 School of Public Health, Sun Yat-sen University, Guangzhou, China; 2 Sun Yat-sen University Global Health Institute, Institute of State Governance, Sun Yat-sen University, Guangzhou, China; 3 Guangdong Key Laboratory of Health Informatics, Guangzhou, China; 4 Research Center of Health Informatics, Sun Yat-sen University, Guangzhou, China; 5 JC School of Public Health and Primary Care, The Chinese University of Hong Kong, Shatin District, Hong Kong, China; 6 The Chinese University of Hong Kong Shenzhen Research Institute, Shenzhen, China; 7 Department of Psychology, Faculty of Social Sciences, University of Macau, Taipa, Macao, China; 8 Department of Rehabilitation Sciences, The Hong Kong Polytechnic University, Hong Kong SAR, China; 9 Mental Health Research Centre, The Hong Kong Polytechnic University, Hong Kong SAR, China; 10 Guangzhou Women and Children’s Medical Center, Guangzhou Medical University, Guangzhou, Guangdong, China; 11 School of Public Health, Guangdong Pharmaceutical University, Guangzhou, China; 12 Acacia Lab for Health Systems Strengthening and Department of Health Management, School of Health Management, Southern Medical University, Guangzhou, China; 13 School of Mental Health, Wenzhou Medical University, Wenzhou, China; 14 Affiliated Kangning Hospital of Wenzhou Medical University, Wenzhou, China; 15 Centre for Health Behaviors Research, The Chinese University of Hong Kong, Shatin District, Hong Kong, China; 16 School of Public Health, Zhejiang University, Hangzhou, China; 17 Center for Global Health Equity, New York University (Shanghai), Shanghai, People’s Republic of China

**Keywords:** COVID-19, healthcare workers, mental health, randomised controlled trials, self-help

## Abstract

**Aims:**

To examine the effectiveness of Self-Help Plus (SH+) as an intervention for alleviating stress levels and mental health problems among healthcare workers.

**Methods:**

This was a prospective, two-arm, unblinded, parallel-designed randomised controlled trial. Participants were recruited at all levels of medical facilities within all municipal districts of Guangzhou. Eligible participants were adult healthcare workers experiencing psychological stress (10-item Perceived Stress Scale scores of ≥15) but without serious mental health problems or active suicidal ideation. A self-help psychological intervention developed by the World Health Organization in alleviating psychological stress and preventing the development of mental health problems. The primary outcome was psychological stress, assessed at the 3-month follow-up. Secondary outcomes were depression symptoms, anxiety symptoms, insomnia, positive affect (PA) and self-kindness assessed at the 3-month follow-up.

**Results:**

Between November 2021 and April 2022, 270 participants were enrolled and randomly assigned to either SH+ (*n* = 135) or the control group (*n* = 135). The SH+ group had significantly lower stress at the 3-month follow-up (*b* = −1.23, 95% CI = −2.36, −0.10, *p* = 0.033) compared to the control group. The interaction effect indicated that the intervention effect in reducing stress differed over time (*b* = −0.89, 95% CI = −1.50, −0.27, *p* = 0.005). Analysis of the secondary outcomes suggested that SH+ led to statistically significant improvements in most of the secondary outcomes, including depression, insomnia, PA and self-kindness.

**Conclusions:**

This is the first known randomised controlled trial ever conducted to improve stress and mental health problems among healthcare workers experiencing psychological stress in a low-resource setting. SH+ was found to be an effective strategy for alleviating psychological stress and reducing symptoms of common mental problems. SH+ has the potential to be scaled-up as a public health strategy to reduce the burden of mental health problems in healthcare workers exposed to high levels of stress.

## Introduction

The coronavirus disease 2019 (COVID-19) pandemic, a major public health emergency, has significantly affected the mental health of healthcare workers globally (Davies *et al.*, 2021; Faria *et al.*, 2021; SeyedAlinaghi *et al.*, 2021; Zoa-Assoumou *et al.*, 2021). The emergence of mental health problems among healthcare workers is exacerbated by various stressors including their high infection risk and long working hours (Zhang *et al.*, 2021). A meta-analysis of 239 studies encompassing 271,319 healthcare workers during the COVID-19 pandemic showed a pooled prevalence of 40% for acute stress, 33% for depression, 42% for anxiety and 42% for insomnia (Aymerich *et al.*, 2022).

Mental health interventions for healthcare workers during public health emergencies are essential (Zaçe *et al.*, 2021). Public health emergencies, especially in developing countries and resource-limited settings, often pose significant challenges and overwhelm mental health systems (Jiang *et al.*, 2020; Luo *et al.*, 2020; Zhang *et al.*, 2020). In addition to a lack of resources, mental health problems are often highly stigmatised in Asian countries, which may also prevent people from seeking help (Clement *et al.*, 2015; Hooper *et al.*, 2021; Shi *et al.*, 2020). Previous systematic review has documented that mindfulness-based interventions (MBIs) have shown promise in alleviating various mental health problems (Goldberg *et al.*, 2018) and reducing stress level, among both clinical and nonclinical populations, particularly in healthcare workers (Lomas *et al.*, 2018; Strauss *et al.*, 2021). However, several barriers hinder the adoption of these interventions among healthcare workers, including lack of availability (Rycroft-Malone *et al.*, 2019), high workplace demands (Mackenzie *et al.*, 2006) and concerns related to stigma (e.g., negative social judgements) (Clement *et al.*, 2015; Taylor *et al.*, 2022). Therefore, there is an urgent need to assess the effectiveness and feasibility of low intensity interventions that do not further burden the overwhelmed health system and can be easily scaled to reach broader populations. Mindfulness-based self-help with MBIs via e-books, online courses and smartphone apps (Taylor *et al.*, 2022) caught attention from researchers. Some interventions have been approved to be effective in reducing mental health problems (Fiol-DeRoque *et al.*, 2021; Taylor *et al.*, 2022). Self-help mental health interventions are one possible solution that can be applied in response to public health emergencies.

In response to the growing demand in self-help psychological support, the World Health Organization (WHO) developed a series of brief transdiagnostic psychological interventions, among them, Self-Help Plus (SH+) – an intervention that is accessible, scalable, easy to use, cost-effective and can be delivered by non-professionals (Purgato *et al.*, 2019). It was trialled in multiple populations such as refugees (Acarturk *et al.*, 2022; Brown *et al.*, 2018; Purgato *et al.*, 2019; Tol *et al.*, 2020) and nursing home workers (Riello *et al.*, 2021). SH+ materials are based on the principles of acceptance and commitment therapy (ACT) (Hayes *et al.*, 2013). ACT combines positive thinking with other acceptance-based practices to help people adapt to difficult thoughts and feelings, cope with stress, be compassionate towards themselves and others and live according to their values (Galante *et al.*, 2021). We identified only one study (Riello *et al.*, 2021) that tested SH+ among healthcare workers, which was conducted with Italian nursing or care home workers during the COVID-19 pandemic. SH+ demonstrated a marginally significant effect (*p* = 0.097) in reducing anxiety and post-traumatic symptomatology (Riello *et al.*, 2021). Further validation of the effects of SH+ in other samples of healthcare workers in different cultural settings is still warranted.

With the normalisation of COVID-19 prevention and control measures, various healthcare workers in China have undertaken tasks including daily or city-wide nucleic acid testing, epidemiological surveys and vaccinations under a tight time schedule (Li *et al.*, 2021c). The stress associated with performing these tasks is enormous. Due to the high workload and lack of mental health services for healthcare workers, there is an urgent need for low-cost, sustainable, effective and flexible mental health interventions (Yang *et al.*, 2021). Moreover, since the pandemic has reduced in-person activities, interventions are needed that can be delivered remotely using internet communication technology and via social media platforms.

In this study, we examined the effectiveness of SH+ for alleviating stress and mental health problems among healthcare workers in China delivered via social networking platforms. We hypothesised that the use of SH+ would result in improvements in indicators of stress and reducing symptoms of common mental health problems at the 3-month follow-up as compared to the control group.

## Methods

### Trial design

This study was a prospective, two-arm, unblinded, parallel-designed randomised controlled trial. The protocol for this study was prospectively registered with the Chinese Clinical Trials Centre (ChiCTR2100052402) and published (Luo *et al.*, 2022). The authors assert that all procedures contributing to this work comply with the ethical standards of the relevant national and institutional committees on human experimentation and with the Helsinki Declaration of 1975, as revised in 2008. All procedures involving human subjects were approved by the Public Health Ethics Committee of Sun Yat-sen University – approval: 2021-120. All adult participants provided electronic informed consent to participate in this study.

### Trial procedure

The participants were recruited from November 30 to 28 December 2021. The intervention was completed on 8 February 2022, and the follow-up was completed by 18 April 2022. Recruitment information was distributed through contact within several types of medical institutions (e.g., general hospitals, community health centres and the Centre for Disease Control and Prevention) in all municipal districts of Guangzhou, Guangdong Province, China. Interested participants provided their appropriate contact information via the QR code on the recruitment literature. Potential participants were then contacted by a research assistant through the social networking platforms account (i.e., WeChat and QQ) they provided.

After the research assistant confirmed the eligibility of potential participants and obtained their informed consent, the participants were invited to complete an online baseline assessment (T0). Follow-up assessments were conducted at 2 weeks (T1), 1 month (T2) and 3 months (T3) after the baseline assessment. The research assistant sent a link to the online questionnaire to all participants via social networking platforms, and the participants were informed that they could complete the questionnaire on any electronic device. All data were collected via an online questionnaire using the Questionnaire Star online survey tool (www.wjx.cn). Upon completion of the baseline and each the follow-up surveys, monetary compensation of Renminbi (RMB) 55 (about US $7.73) in total was provided to the participants for their time. A gift with equivalent value of RMB 80 (about US $11.24) was provided to the participants who completed all follow-up assessments.

### Randomisation and masking

The participants were randomly assigned either to the intervention or the control group in a block randomisation (size = 4), in a 1:1 ratio. Randomisation was performed using a random number generator. The allocation results were known to the researchers. As the participants had either direct access to the SH+ exercise or had a waiting period (i.e., receiving control materials but with the option to use SH+ after the study), they knew their group assignment. The participants in the control group were informed that they would receive the SH+ materials 3 months after the start of the project.

### Inclusion and exclusion criteria

Participants were included if they met the following criteria: (a) 18 years of age or older; (b) currently a healthcare worker (including in the clinical, nursing and public health fields); (c) exceeded the 15-point threshold on the 10-item Perceived Stress Scale (PSS-10) which suggests a high level of stress (Cohen *et al.*, 1983; Weiner *et al.*, 2020); (d) able to complete the online questionnaire independently and (e) had a mobile communication device that was connected to the internet.

The exclusion criteria were the following: (a) having a severe mental problem or suicidal ideation; (b) being unreachable after 3 days of attempts at different times via the contact information given; and (c) planning to leave Guangzhou frequently for business in the next month. Those with severe mental health problems or suicidal ideation were referred to psychiatric professional institutes.

### Experimental and control intervention

#### Intervention group

The intervention was a web-based self-managed stress management programme called SH+. SH+ was developed by the WHO and collaborators working in mental health and psychosocial support in humanitarian settings (Epping-Jordan *et al.*, 2016). A Chinese version was approved and published by the WHO (Yang *et al.*, 2021). SH+ intervention materials have two components: audio recordings and an accompanying illustrated manual. These materials were divided into seven exercises in this study (Epping-Jordan *et al.*, 2016). The contents of SH+ were shown in Table S1. During the 1-month intervention period, the two SH+ exercises were distributed to the participants weekly (World Health Organization, 2020), following the intervention frequency and duration recommended according to a systematic review of ACT intervention studies. Studies showed that social networking systems can serve as a platform for mental health interventions while reducing implementation costs and increasing their effectiveness (Li *et al.*, 2021b; Yang *et al.*, 2020). The SH+ was delivered via QQ or WeChat, the two most popular social network platforms in China. Each exercise took approximately 10 minutes to complete. A gift with equivalent value of RMB 80 (about US $11.24) was provided to the participants completed all exercises. Details of the intervention programme and schedule were described in the published protocol (Luo *et al.*, 2022).

#### Control group

During the 1-month intervention period, participants in the control group received weekly messages about mental health promotion via their personal social networking accounts. For example, the initial message delivered in the first week was psychoeducation around stress and mental health, the main factors affecting mental health and a description of positive mental health; for the second week, the message was an introduction to the link between physical and mental health. These materials were developed by a psychologist and were described in detail in the protocol (Luo *et al.*, 2022).

### Outcomes

All assessments were completed online via self-reported questionnaires. All outcome measures and their corresponding time points were described in Table S2.

## Primary outcome

### Stress

The PSS-10 was used to measure participant-reported stressful events that occurred in the past month (Cohen *et al.*, 1983). The scale consists of 10 items scored on a 5-point Likert scale: 0, never; 1, almost never; 2, occasionally; 3, sometimes and 4, often. The total PSS-10 score ranged from 0 to 40, with higher scores indicating higher levels of stress. The Chinese version of the scale has been shown to have good reliability and validity (Leung *et al.*, 2010). Reliability of the scale was excellent in the current study (Cronbach’s alpha = 0.863). Stress was assessed at all follow-up time points.

## Secondary outcomes

### Depression

Depressive symptoms in the previous 2 weeks were measured using the Chinese version of the 9-item Patient Health Questionnaire (PHQ-9) Depression Scale (Spitzer *et al.*, 1999). The scale is scored using a 4-point Likert scale: 0, not at all; 1, rarely; 2, many times and 3, almost every day. The total score of the PHQ-9 ranges from 0 to 27, with a score of 10 usually considered as the cut-off point for having significant depressive symptoms (Kroenke *et al.*, 2010). The Chinese version of the PHQ-9 scale has been shown to have good reliability and validity (Wang *et al.*, 2014).

### Anxiety

Anxiety symptoms in the previous 2 weeks were assessed using the Chinese version of the Generalised Anxiety Disorder-7 (GAD-7) Scale (Spitzer *et al.*, 2006). The scale has seven items scored on a 4-point Likert scale: 0, not at all; 1, rarely; 2, many times and 3, almost every day, with a total score between 0 and 21. A score of 10 on this scale is usually considered the cut-off point for having significant anxiety (Spitzer *et al.*, 2006). The Chinese version of the GAD-7 scale has been shown to have good reliability and validity (Tong *et al.*, 2016).

### Insomnia

Insomnia was assessed using the Insomnia Severity Index, a seven-item scale with each item rated from 0 to 4 (Morin *et al.*, 2011). The scale has a total score of 28, with scores of 0–7 indicating no insomnia, 8–14 indicating subclinical insomnia, 15–21 indicating moderate insomnia and 22–28 indicating severe insomnia (Wong *et al.*, 2017a). The scale has been shown to have good reliability and validity in the Chinese population (Wong *et al.*, 2017a).

### Job burnout

Job burnout was assessed using one self-constructed item (i.e., ‘I feel exhausted from work.’) scored on a 7-point Likert scale in which 0 indicates ‘never’ and 6 indicates ‘every day’. Higher scores indicate more burnout. A score of two or less is labelled as no burnout, three and four are labelled as mild to moderate burnout and five and above are labelled as severe burnout.

### Positive affect (PA)

PA was assessed using the PA subscale of the Positive and Negative Affect Scale (PANAS) (Watson *et al.*, 1988). The PA subscale contains five emotion descriptors with the following five response options: almost none, relatively little, moderately, more and very strongly corresponding to 1, 2, 3, 4 and 5, respectively. The total PA score ranges from 5 to 25, with higher scores indicating a more positive emotional experience (Watson *et al.*, 1988). The PANAS has good reliability and validity in the Chinese population (Li *et al.*, 2020).

### Self-kindness

Self-kindness was measured using the self-kindness subscale of the 26-item Self-Compassion Scale (SCS) (Neff, 2003). The subscale has five items which were scored on a 5-point Likert-type scale, with responses ranging from 1, very unlikely to 5, very likely. Total scores were calculated with higher scores indicating that individuals can treat themselves better when dealing with adversity (Neff, 2003). Previous research, including studies with Chinese youth, has shown that the SCS has good reliability and validity (Li *et al.*, 2021a).

PA and self-kindness were assessed at baseline (T0) and 1-month (T2) and 3-month (T3) follow-up, and other secondary outcomes were assessed only at baseline and 3-month follow-up.

## Process assessment and intervention adherence

Information was collected about the potential contamination (e.g., ‘During the intervention, did you subscribe to any other resources that provided information about stress?’), perceived effectiveness of the intervention (e.g., ‘Do you think this programme is effective in relieving stress?’), compliance with the self-help stress intervention (e.g., ‘How much of the seven intervention materials were you able to seriously complete?’) and perceptions towards and experiences in using SH+ (e.g., ‘Will you actively use SH+ to help you relieve stress in the future?’ and ‘Do you think the intervention materials presented in this project are easy to do?’). These questions were rated on a 5-point Likert scale. For example, the compliance was rated from 0 ‘not at all’ to 4 ‘every time’. For each exercise, a question embedded in the materials was used to test whether the participant had completed the intervention conscientiously.

## Covariates

Social-demographic information and work-related variables included gender, age, education level, marital status, monthly income, number of people in the home, physical exercise, alcohol use, type of workplace, years of employment, job title, weekly working hours and job burnout. Physical exercise was measured using the Physical Activity Rating Scale-3 (Liang, 1994). Alcohol use was measured using the three-item Alcohol Use Disorders Identification Test-Concise (Saunders *et al.*, 1993).

## Sample size estimates

Previous studies on the effectiveness of ACT-based interventions in reducing stress have shown an effect size of 0.63 (Wersebe *et al*., 2018). Given the standard deviation of 4.95 (Wersebe et al., 2018), a reduction of 3.12 (0.63 × 4.95 = 3.12) in PSS stress score is expected. The sample size of the study was calculated using an efficacy of 90% and an alpha level of 0.05 in a two-tailed test. We used Power Analysis and Sample Size software (NCSS, Kaysville, UT, USA) to calculate the required sample size of 108 (54 per group). The final targeted sample size was 216 (108 per group) assuming a 50% adherence rate at month 3 of follow-up based on previous data (Geary and Rosenthal, 2011).

## Statistical analysis

The chi-square test was used for categorical variables, while the student’s *t*-test was used for continuous variables to test for between-group differences at baseline and follow-up time points. Paired *t*-tests were conducted for within-subject analyses to compare baseline responses with follow-up responses. We used an intention-to-treat approach to analyse primary and secondary outcomes. To examine the overall effect of the intervention group versus the control group while controlling for potential covariates, repeated measures analyses were performed by using generalised estimating equations (GEEs) (Zorn, 2001). First, we entered time and the main effect of the intervention in the model (Model 1); we then entered time, the main effect of the intervention and baseline outcome scores (Model 2); in Model 3, we added an interaction term between intervention condition and time to Model 2. We performed multiple imputation to address missing data and conducted analyses using GEE model on the imputed dataset. All statistical tests were two-sided, and *p* < 0.05 was considered statistically significant. For sensitivity analyses, we used ANOVA trend test to assess the intervention effects by various compliance levels. All data analyses were conducted using R4.1.2.

## Results

**Figure 1.**
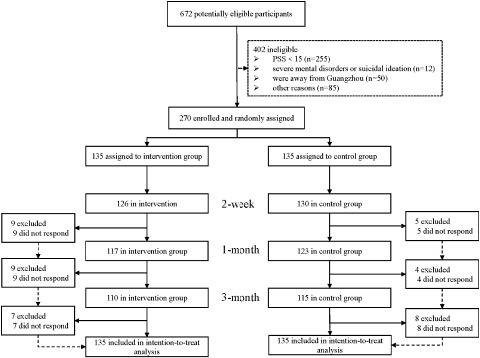
The CONSORT flow diagram of the study.

After screening 672 potentially eligible participants, a total of 402 participants were excluded from the study. Among them, 255 were excluded due to their stress levels falling below the established cut-off, 12 participants had self-reported severe mental health problems or suicidal ideation, 50 participants were expected to be away from Guangzhou during the study period and 89 participants were excluded for other reasons (i.e., cannot be reached through the provided contact information). The remaining 270 individuals who met the inclusion criteria and agreed to take part in the study were randomly assigned to either the intervention group (*n* = 135) or the control group (*n* = 135). At the 3-month follow-up, 25 (18.5%) participants in the intervention group and 20 (14.8%) participants in the control group were lost to follow-up due to non-contact (see Fig. 1 for details). Except for monthly income, all baseline characteristics were balanced between the 45 participants who were lost to follow-up and the 225 who completed all follow-up surveys (Table S3).

## Baseline background characteristics of the participants

The main baseline characteristics were similar between the intervention group and the control group (Table 1). In terms of socio-demographics, 72.6% of the participants were below 35 years old; 71.9% were female; 72.2% lived in the four older districts of Guangzhou (e.g., Yuexiu District); 83.7% had a bachelor’s degree or higher; 45.6% were married; 79.3% lived with others and half had a monthly income of RMB 5000–10,000. In terms of work-related variables, most participants (84.1%) worked in hospitals or community health centres; 62.6% had a junior job title or below; 65.5% worked more than 40 hours per week. In terms of mental health problems, 47.0% had mild to moderate job burnout, and 64.1% had significant depressive symptoms and 48.5% had significant anxiety symptoms.

**Table 1.** Baseline characteristics of the participants in the intervention and control groups

Variable	All (*n* = 270) *N* (%)	Control (*n* = 135) *N* (%)	Intervention (*n* = 135) *N* (%)	*p*
Gender				
Male	76 (28.1)	41 (30.4)	35 (25.9)	0.417^a^
Female	194 (71.9)	94 (69.6)	100 (74.1)	
Age (years)				
<35	196 (72.6)	97 (71.9)	99 (73.3)	0.785^a^
≥35	74 (27.4)	38 (28.1)	36 (26.7)	
District				
Four older districts (e.g., Yuexiu)	195 (72.2)	92 (68.1)	103 (76.3)	0.135^a^
Other districts (e.g., Nansha)	75 (27.8)	43 (31.9)	32 (23.7)	
Education level				
Below bachelor’s degree	44 (16.3)	27 (20.0)	17 (12.6)	0.099^a^
Bachelor’s degree or above	226 (83.7)	108 (80.0)	118 (87.4)	
Marital status				
Single/widow/other	147 (54.4)	69 (51.1)	78 (57.8)	0.271^a^
Married	123 (45.6)	66 (48.9)	57 (42.2)	
Living situation				
Living alone	56 (20.7)	30 (22.2)	26 (19.3)	0.548^a^
Living with others	214 (79.3)	105 (77.8)	109 (80.7)	
Monthly income (Yuan)				
<5000	66 (24.4)	34 (25.2)	32 (23.7)	0.944^a^
5000−10,000	130 (48.2)	65 (48.1)	65 (48.1)	
>10,000	74 (27.4)	36 (26.7)	38 (28.2)	
Alcohol use				
Yes	138 (51.1)	64 (47.4)	74 (54.8)	0.233^a^
No	132 (48.9)	71 (52.6)	61 (45.2)	
Physical exercise				
No exercise	132 (48.9)	57 (42.2)	75 (55.6)	0.134^a^
Low exercise	92 (34.1)	54 (40.0)	38 (28.1)	
Medium exercise	34 (12.6)	17 (12.6)	17 (12.6)	
High exercise	12 (4.4)	7 (5.2)	5 (3.7)	
Type of workplace				
Hospital	101 (37.4)	45 (33.3)	56 (41.5)	0.365^a^
Community health centre	126 (46.7)	68 (50.4)	58 (43.0)	
Centres for Disease Control and Prevention	43 (15.9)	22 (16.3)	21 (15.5)	
Job title				
Junior and below	169 (62.6)	82 (60.7)	87 (64.4)	0.529^a^
Intermediate and above	101 (37.4)	53 (39.3)	48 (35.6)	
Years of employment				
<3	71 (26.3)	29 (21.5)	42 (31.1)	0.169^a^
3−9	107 (39.6)	55 (40.7)	52 (38.5)	
≥10	92 (34.1)	51 (37.8)	41 (30.4)	
Weekly hours of work				
≤40	93 (34.4)	39 (28.9)	54 (40.0)	0.055^a^
>40	177 (65.6)	96 (71.1)	81 (60.0)	
Job burnout				
No burnout	82 (30.4)	37 (27.4)	45 (33.3)	0.242^a^
Mild to moderate burnout	127 (47.0)	62 (45.9)	65 (48.1)	
Severe burnout	61 (22.6)	36 (26.7)	25 (18.5)	
Significant depression symptoms				
No	97 (35.9)	48 (35.6)	49 (36.3)	0.899^a^
Yes	173 (64.1)	87 (64.4)	86 (63.7)	
Significant anxiety symptoms				
No	139 (51.5)	70 (51.9)	69 (51.1)	0.903^a^
Yes	131 (48.5)	65 (48.1)	66 (48.9)	
Insomnia				
None or subclinical insomnia	217 (80.4)	107 (79.3)	110 (81.5)	0.646^a^
Moderate to severe insomnia	53 (19.6)	28 (20.7)	25 (18.5)	
Stress mean (SD)	21.29 (4.43)	21.34 (4.60)	21.23 (4.26)	0.837^b^
Depression mean (SD)	11.48 (4.42)	11.54 (4.60)	11.42 (4.26)	0.826^b^
Anxiety mean (SD)	9.54 (4.01)	9.44 (4.47)	9.64 (3.50)	0.672^b^
Insomnia mean (SD)	10.27 (5.47)	10.16 (5.54)	10.38 (5.41)	0.739^b^
Job burnout mean (SD)	3.33 (1.36)	3.43 (1.41)	3.24 (1.31)	0.245^b^
Positive effect mean (SD)	25.43 (5.64)	25.89 (6.01)	24.97 (5.21)	0.181^b^
Self-kindness mean (SD)	16.86 (3.83)	16.99 (3.89)	16.72 (3.77)	0.557^b^

a The chi-square test was used for categorical variables.

b The student’s *t*-test was used for continuous variables.

### Primary outcome

**Figure 2.**
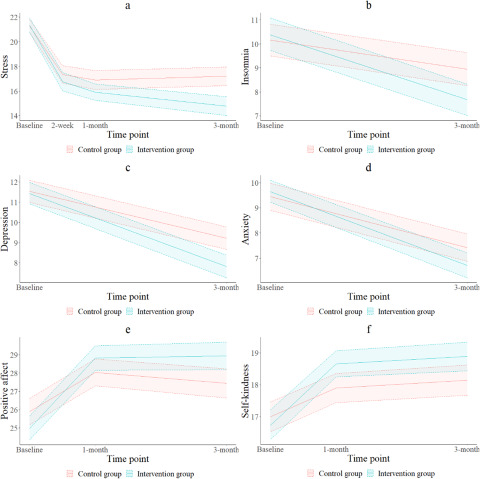
Trend of primary and secondary outcomes between the intervention and control groups*.*a: Stress; b: Insomnia; c: Depression; d: Anxiety; e: Positive affect; f: Self-kindness. Primary outcome (stress) was assessed at all time points. Positive affect and self-kindness were assessed at T0, T2, and T3, and other secondary outcomes wereassessed only at T0 and T3.

Univariate comparisons showed that the SH+ group demonstrated a statistically significant difference from the control group in changes from the baseline score on PSS, indicating that SH+ was effective in reducing perceived stress compared with the control at the 3-month follow-up (the decrease in PSS at the 3-month follow-up (T3) from baseline was 6.34 ± 7.03 for SH+ vs. 4.23 ± 6.48 for control group, *p* = 0.020, Table 2). However, significant differences were not detected at the 2-week follow-up (T1) or the 1-month follow-up (T2). The GEE analysis also showed a significant main effect of SH+ versus control in reducing stress over the study period (regression coefficient *b* = −1.23, 95% CI = −2.36, −0.10, *p* = 0.033, model 2 in Table 3), when controlling for baseline stress score. The interaction effect between the intervention and time was also statistically significant, indicating that the intervention effect in reducing stress differed over time (*b* = −0.89, 95% CI = −1.50, −0.27, *p* = 0.005, model 3 in Table 3). This interaction effect can also be observed in Fig. 2a that the intervention effect was larger at the 3-month follow-up as compared to the 2-week and 1-month follow-up. Approximately, 85% of the participants participated in SH+ exercises at different frequencies over the course of 1 month of intervention (Table S6). In addition, the results of the 1-month trend test showed that as the frequency of practicing SH+ increased, the changes in stress score from baseline increased (Table S4). Same results were observed for the 3-month trend test (Table S5). The results of the sensitivity analysis using the data after multiple imputation were generally consistent with the present results (Table S7).

**Table 2.** Comparison of between-group and within-group differences in primary and secondary outcomes for the intervention and control groups over the study period

	Control group		Intervention group		Between-group difference	
Variable	Mean (SD)	Change from T0, mean (SD)^d^	Mean (SD)	Change from T0, mean (SD)^d^	*p*^a^^,^^c^	*P*^b^^,^^c^
Primary outcome^e^						
Stress						
T1^g^	17.35 (5.48)	−4.11 (5.94)***	16.75 (5.65)	−4.42 (6.54)***	0.383	0.689
T2^h^	16.89 (5.81)	−4.59 (6.20)***	15.91 (5.07)	−5.37 (6.44)***	0.162	0.344
T3^i^	17.20 (5.81)	−4.23 (6.48)***	14.77 (5.84)	−6.34 (7.03)***	0.002	0.020
Secondary outcomes^f^						
Depression						
T3	9.22 (4.27)	−2.17 (4.34)***	7.81 (4.31)	−3.47 (5.23)***	0.015	0.043
Anxiety						
T3	7.42 (4.11)	−1.92 (4.46)***	6.71 (3.78)	−2.83 (4.52)***	0.180	0.128
Insomnia						
T3	8.95 (5.23)	−1.23 (5.93)***	7.67 (5.02)	−2.80 (4.89)***	0.063	0.031
Burnout						
T3	3.03 (1.40)	−0.34 (1.62)*	2.84 (1.23)	−0.39 (1.52)**	0.261	0.805
Positive affect						
T2	28.04 (5.65)	2.22 (5.75)***	28.82 (5.12)	3.68 (5.43)***	0.265	0.046
T3	27.44 (6.12)	1.66 (6.11)***	28.94 (5.76)	3.65 (5.54)***	0.061	0.010
Self-kindness						
T2	17.89 (3.51)	0.82 (3.49)*	18.66 (3.10)	1.87 (3.08)***	0.076	0.014
T3	18.14 (3.60)	1.06 (4.31)**	18.88 (3.43)	2.03 (3.40)***	0.115	0.048

a Mean differences between groups.

b Difference in change from T0 between groups.

c Student’s *t*-test.

d Paired *t*-test.

e Primary outcome was assessed at all time points.

f Positive affect and self-kindness were assessed at T0, T2 and T3, and other secondary outcomes were assessed only at T0 and T3.

g There were 130 and 126 participants in the control group and intervention group, respectively.

h There were 123 and 117 participants in the control group and intervention group, respectively.

i There were 115 and 110 participants in the control group and intervention group, respectively.

* *p* < 0.05; **p < 0.01; ***p < 0.001.

**Table 3.** Generalised estimating equations predict stress at different time points and other secondary outcomes

	Model 1		Model 2		Model 3	
Variable	*β* (95% CI)	*p*	*β* (95% CI)	*p*	*β* (95% CI)	*p*
Primary outcome						
**Stress**						
Group						
Control	Ref	0.030	Ref	0.033	Ref	0.567
Intervention	−1.33 (−2.52, −0.13)		−1.23 (−2.36, −0.10)		0.48 (−1.15, 2.10)	
Time	−0.46 (−0.77, −0.15)	0.004	−0.46 (−0.77, −0.15)	0.004	−0.03 (−0.46, 0.41)	0.901
Baseline stress	Na	Na	0.29 (0.13, 0.45)	<0.001	0.29 (0.13, 0.45)	<0.001
Group*Time	Na	Na	Na	Na	−0.89 (−1.50, −0.27)	0.005
Secondary outcome						
**Self-kindness**						
Group						
Control	Ref	0.026	Ref		Ref	
Intervention	0.86 (0.10, 1.61)		0.87 (0.23, 1.50)	0.007	0.83 (−1.22, 2.87)	0.428
Time	0.26 (−0.16, 0.67)	0.222	0.24 (−0.17, 0.66)	0.250	0.23 (−0.36, 0.82)	0.436
Baseline self-kindness	Na	Na	0.43 (0.33, 0.54)	<0.001	0.43 (0.33, 0.54)	<0.001
Group*Time	Na	Na	Na	Na	0.02 (−0.81, 0.84)	0.968
**Positive affect**						
Group						
Control	Ref	0.021	Ref		Ref	
Intervention	1.41 (0.21, 2.62)		1.57 (0.50, 2.64)	0.004	−0.27 (−3.18, 2.64)	0.856
Time	−0.20 (−0.78, 0.37)	0.490	−0.22 (−0.80, 0.36)	0.455	−0.58 (−1.38, 0.21)	0.151
Baseline positive affect	Na	Na	0.43 (0.31, 0.56)	<0.001	0.43 (0.31, 0.56)	<0.001
Group*Time	Na	Na	Na	Na	0.74 (−0.40, 1.89)	0.204

Na = not applicable.

## Secondary outcomes

Table 2 shows that compared to the control group, the SH+ group had statistically significant improvements in most of the secondary outcomes, including depression, insomnia, PA and self-kindness at 3-month follow-up. For example, the increases in the PA scale from baseline were 3.65 for SH+ versus 1.66 for the control group (*p* = 0.010). The GEE analysis showed that after controlling for baseline self-kindness scores, the SH+ group (*b* = 0.87, 95% CI = 0.23, 1.50, *p* = 0.007, model 2 in Table 3) had a statistically significant increase in self-kindness as compared to the control group. Similarly, after controlling for baseline PA scores, the main effect of SH+ in increasing PA (*b* = 1.57, 95% CI = 0.50, 2.64, *p* = 0.004, model 2 in Table 3) was also statistically significant. There were no interaction effects between time and intervention for self-kindness (*b* = 0.02, 95% CI = −0.81, 0.84, *p* = 0.968, model 3 in Table 3) and PA (*b* = 0.74, 95% CI = −0.40, 1.89, *p* = 0.204, model 3 in Table 3). In addition, the results of the 3-month trend test showed that as the frequency of practicing SH+ increased, the changes in depression, insomnia and PA score from baseline increased (Table S5). The results of the sensitivity analysis using the data after multiple imputation were generally consistent with the present results (Table S7).

## Intervention compliance and process evaluation

The compliance with the interventions was generally satisfactory. Of the 110 participants in the intervention group who completed three follow-up surveys, 100 (90.9%) conducted the SH+ exercises most of the time during the intervention. Over 85% of the participants continued to practice SH+ exercises with varying frequency even after the 1-month intervention period. The majority, 85 (77.3%) perceived the SH+ exercises to be effective in reducing stress, and 86 (78.1%) reported that they would actively use the exercises occasionally or regularly thereafter to reduce stress after the intervention. In addition, 105 (95.4%) and 103 (93.6%) participants, respectively, found the SH+ intervention materials were easy to understand and use. In the study period, there were no significant adverse events. The details are shown in Table 4.

**Table 4.** Intervention group completion effect evaluation at 3 months (*n* = 110)

Items	*N* (%)
How would you rate your completion of the seven intervention materials in this project?	
Carefully completed every time	65 (59.1)
Mostly completed carefully	35 (31.8)
Completed about half of the time	7 (6.4)
Completed a small part of the time	3 (2.7)
What is the status of your use of our intervention materials since the end of the intervention period to date?	
No practice (0 times)	16 (14.5)
Occasionally practice (1 time/month)	53 (48.2)
Sometimes practice (2–3 times/month)	36 (32.7)
Practice often (1–4 times/week)	5 (4.5)
Do you think this programme is effective in relieving stress?	
Very effective	10 (9.1)
Somewhat effective	75 (68.2)
Fairly effective	19 (17.3)
Not very effective	16 (14.6)
Will you actively use the methods of this programme to help you relieve stress in the future?	
Will continue to use it regularly	27 (24.5)
Will use it occasionally	59 (53.6)
Not sure if I will use it	22 (20.0)
Not likely to continue to use it	2 (1.8)
Did you find the intervention materials promoted by the programme easy to understand?	
Particularly easy	38 (34.5)
Relatively easy	67 (60.9)
Fairly easy	4 (3.6)
Not very easy	1 (0.9)
Do you think the intervention materials presented in this project are easy to do?	
Particularly easy	26 (23.6)
Relatively easy	77 (70.0)
Fairly easy	6 (5.5)
Not very easy	1 (0.9)

## Discussion

This study evaluated the effectiveness of the WHO SH+ programme in reducing stress and mental health problems among healthcare workers in China during the COVID-19 pandemic utilising a randomised controlled trial. The participants in SH+ showed improvements in stress, self-kindness and PA over the study period. We also detected significantly fewer depression symptoms among SH+ participants compared with the control group at 2 months post-intervention. A dose–response relationship was also observed with greater SH+ intervention compliance being significantly associated with improved stress and mental health outcomes.

This study found that SH+ was effective in alleviating stress and improving mental health among healthcare workers. This corroborates the results of previous efficacy studies of SH+ among refugee populations (Acarturk *et al.*, 2022; Tol *et al.*, 2020). To the best of our knowledge, although this study was not the first application of SH+ for healthcare workers, it was the first study conducted among this population in a low-resource setting and with positive trial results. In Riello’s trial (Riello *et al.*, 2021) of SH+ among Italian healthcare workers, SH+ showed marginally but not statistically significant effect in reducing anxiety and post-traumatic symptoms. The effect of SH+ in reducing anxiety was also not significant in our sample, which is consistent with Riello’s study. It suggests that while SH+ is a transdiagnostic treatment, it may not consistently reduce anxiety. This current study fills a research gap and provides empirical evidence for the effectiveness of SH+ in healthcare workers in a different setting: first, the Italian study was limited to a population of nursing and care home workers, while the present study had a more diverse and general sample of healthcare workers including both clinical workers and public health workers; second, in Riello’s study, they included not only healthcare workers but also administrative and technical staff from nursing and care homes, the overall education level was lower than that of the current study. The most frequent highest educational level was the high school (46.64%) in Riello’s study versus 83.7% participants in the current study had a bachelor’s degree or higher. Previous studies suggested that participants with higher educational levels had higher intervention effects (Warmerdam *et al.*, 2013) and fewer dropouts (Spek *et al.*, 2008) for internet-based interventions; third, the dosage and compliance with the intervention may vary in different settings and populations. Over 90% of the SH+ participants in the present study self-reported that they completed the exercises most of the time, while the compliance was expected to be lower in Riello’s study since 20% participants never logged in their study site.

Compared with traditional psychological interventions that are delivered by specialist providers, this SH+ intervention can be delivered to participants via their mobile device, making it more accessible in low-resource settings with a large shortage of psychiatrists and clinical psychologists. It also has several advantages being delivered through social networking platforms making treatment efficient and delivered in standardised manner, as intended, without the need for extensive manpower. During the COVID-19 pandemic, especially in the context of strict epidemic control in China, many healthcare workers have been chronically stressed and need simple and effective strategies to improve their mental health (Li *et al.*, 2021c; Zhang *et al.*, 2021). During the data collection period of this study, in April 2022 (Guangzhou Center for Disease Control and Prevention, 2023), there were two major COVID-19 waves in Guangzhou yet we still observed significant improvements in the primary and some secondary outcomes for the SH+ group, implying that SH+ would be an ideal mental health intervention for healthcare workers given the possibility for multiple COVID-19 waves and other infectious disease pandemics in the future. Considering the abovementioned advantages and evidence from this study, we conclude that SH+ is a promising tool to improve mental health of larger populations in adverse circumstances, especially among healthcare workers during the COVID-19 pandemic for extended periods of intense and stressful work.

Although SH+ is expected to be a scalable and evidence-based stress intervention for populations affected by adversity (World Health Organization, 2017), implementation studies are still needed to address the ‘know-do’ gap in real world settings. When implemented at large scale, SH+ may become less effective than it was in this trial. One example of an implementation challenge would be how to ensure a satisfactory compliance with SH+. In the current efficacy study, we set up a reward mechanism and a reminder service, and the participants can easily communicate with the research team via social networking platform. Thus, the compliance and retention rate (over 90%) were high as compared to 50% from previous web-based interventions (Geary and Rosenthal, 2011). Hybrid type 1 trials are warranted to assess implementation effectiveness of SH+. Other implementation challenges may include staff training and supervision, financing and organisational factors. Implementation studies are needed to assess the optimal strategies to expand the intervention to existing public services and systems in different settings (Proctor *et al.*, 2009).

We note several limitations of the study. First, this study employed convenience sampling, recruiting participants mainly through social networking platform posters. This approach could potentially impact the external validity of our findings. The 2021 Guangzhou City Health Statistics Yearbook (Guangzhou Municipal Health Commission, 2023) showed that 71.1% of Guangzhou healthcare workers were female and 28.9% were male, and 48.7% were under the age of 35. Regarding the distribution of job titles, 67.9% were junior and below and 32.1% were intermediate and senior. Regarding the distribution of education level, 85.7% were bachelor’s degree or above and 14.3% were below bachelor’s degree. According to those statistics, the distribution of key socio-demographic characteristics in our sample is similar to that among healthcare workers in Guangzhou city, except for a slightly younger age distribution. Second, due to the nature of the content of the intervention, a double-blind design was not feasible. Like most Randomised Control Trials (RCTs) of psychological interventions, this study also used an open study design, such that the participants were not blinded to the intervention. Participants in the control group were aware that they would receive SH+ at 3 months after the baseline assessment. However, both participants and research assistants were instructed not to circulate any intervention materials received during the study. Third, the limitations of the selection of the study population may affect the generalisability of the results to the general population, and further RCTs of SH+ for other populations with longer follow-up period may be needed in the future. Another limitation of the study is the reliance on self-report measures for assessing the fidelity of the intervention. While self-report measures are commonly used in psychological research, they are subject to certain constraints. Participants may provide socially desirable responses or align their answers with study expectations, leading to potential response biases. Furthermore, though the difference in scores between the two groups of the PSS at endpoint is statistically significant, it remains unclear whether it is clinically relevant, as the change of 6.34 from T0 in intervention group was a little lower than those in recent studies (Almen *et al.*, 2020; Leth-Nissen *et al.*, 2023). Previous studies in effectiveness of SH+ also showed the limited reduction on perceived stress (Tol *et al.*, 2020). The effectiveness of SH+ might be overstated. Finally, we were not able to control all confounding factors that might have affected the study results, although we were able to adjust for several potential confounding variables in the current study. Future trials could include multicentre approach or conduct cluster randomisation of health centres, different cities, and in rural and urban settings, to assess generalisability.

## Conclusion

SH+ is effective among Chinese healthcare workers to reduce stress and depression symptoms and increase self-kindness and PA. This self-help psychological intervention is sustainable and effective and has the potential to expand to large populations with limited access to social and health services but with a high degree of need. Implementation research and cost-effective analysis are warranted and should be integrated within effectiveness trials in the future to speed the progression of SH+ towards widespread use.

## Supporting information

Li et al. supplementary material Li et al. supplementary material

## Data Availability

The data are not publicly available due to confidentiality and ethical considerations. Deidentified data are available from the authors upon reasonable request and subject to approval by the ethics committees overseeing the study.

## Supplementary material

The supplementary material for this article can be found at https://doi.org/10.1017/S2045796024000106.
