# Supplementary material for: Effectiveness of a WHO self-help psychological intervention to alleviate stress among healthcare workers in the context of COVID-19 in China: a randomised controlled trial
Source: Epidemiol Psychiatr Sci. 2024 Mar 7;33:e11. doi: 10.1017/S2045796024000106 (PMC10940054; doi:10.1017/S2045796024000106)
Supplement: Li et al. supplementary material [file S2045796024000106sup001.docx]

**Table S1**. **The content of the Internet-based self-help stress management intervention**

| Topic | Content |
| --- | --- |
| 1. Building awareness | Learn ways to engage in your life or focus on something you are doing in the moment. |
| 2. Grounding exercise 1 | Learn how to “ground” yourself when experiencing an emotional storm. |
| 3. Grounding exercise 2 | Learn more about how to “ground” yourself when experiencing an emotional storm. |
| 4. Notice and name | Learn how to eliminate negative emotions and thoughts that cause stress. |
| 5. Acting on your values | Learn how to bring your actions closer to your values when under stress. |
| 6. Being kind | Learn ways to be kind to others and yourself, despite adversity. |
| 7. Making room | Learn ways to make room for thoughts and feelings that bother you, without being hurt by them. |

**Table S2. Assessment schedule**

|  | **Study Period** | | | |
| --- | --- | --- | --- | --- |
|  | **Enrollment** | **Follow-up** | | |
| **Time Point** | **T0**  **Baseline** | **T1**  **2 weeks** | **T2**  **1 month** | **T3**  **3 months** |
| Primary outcome variable |  |  |  |  |
| Stress | **×** | **×** | **×** | **×** |
| Secondary outcome variables |  |  |  |  |
| Depressive symptoms | **×** |  |  | **×** |
| Anxiety symptoms | **×** |  |  | **×** |
| Insomnia | **×** |  |  | **×** |
| Positive affects | **×** |  | **×** | **×** |
| Self-kindness | **×** |  | **×** | **×** |
| Baseline variables |  |  |  |  |
| Sociodemographic characteristics | **×** |  |  |  |
| Work-related information | **×** |  |  |  |
| Physical exercise | **×** |  |  |  |
| Alcohol use | **×** |  |  |  |
| Process evaluation |  |  |  |  |
| Intervention utilization |  | **×** | **×** | **×** |
| Intervention adherence |  | **×** | **×** | **×** |
| Contamination |  | **×** | **×** | **×** |

**Table S3**. **Differences in Baseline Characteristic Variables Between Those Who Completed Three Follow-Ups and Those Lost to Follow-Up***

| Variable | n = 270 | | |
| --- | --- | --- | --- |
|  | Three follow-ups completed (n = 225) | Lost to follow-up (n = 45) | *p* |
| Gender |  |  |  |
| Men | 59 (26.2) | 17 (37.8) | 0.116 |
| Women | 166 (73.8) | 28 (62.2) |  |
| Age (years) |  |  |  |
| <35 | 164 (72.9) | 32 (71.1) | 0.807 |
| ≥35 | 61 (27.1) | 13 (28.9) |  |
| District |  |  |  |
| Four older districts (e.g., Yuexiu) | 166 (73.8) | 29 (64.4) | 0.202 |
| Other districts (e.g., Nansha) | 59 (31.9) | 16 (35.6) |  |
| Education level |  |  |  |
| Below bachelor’s degree | 39 (17.3) | 5 (11.1) | 0.302 |
| Bachelor’s degree or above | 186 (82.7) | 40 (88.9) |  |
| Marital status |  |  |  |
| Single/Widow/Other | 125 (55.6) | 22 (48.9) | 0.412 |
| Married | 100 (44.4) | 23 (51.1) |  |
| Living situation |  |  |  |
| Living alone | 48 (21.3) | 8 (17.8) | 0.591 |
| Living with others | 177 (78.7) | 37 (82.2) |  |
| Monthly income (yuan) |  |  |  |
| <5000 | 53 (23.6) | 13 (28.9) | 0.033 |
| 5000-10000 | 116 (51.6) | 14 (31.1) |  |
| >10000 | 56 (24.9) | 18 (40.0) |  |
| Alcohol use |  |  |  |
| Yes | 114 (50.7) | 24 (53.3) | 0.744 |
| No | 111 (49.3) | 21 (46.7) |  |
| Physical exercise |  |  |  |
| No exercise | 114 (50.7) | 18 (40.0) | 0.364 |
| Low exercise | 74 (32.9) | 18 (40.0) |  |
| Medium exercise | 26 (11.6) | 8 (17.8) |  |
| High exercise | 11 (4.9) | 1 (2.2) |  |
| Type of workplace |  |  |  |
| Hospital | 86 (38.2) | 15 (33.3) | 0.612 |
| Community health centre | 102 (45.3) | 24 (53.3) |  |
| Centres for Disease Control and Prevention | 37 (16.4) | 6 (13.3) |  |
| Job title |  |  |  |
| Primary and below | 145 (64.4) | 24 (53.3) | 0.160 |
| Intermediate and above | 80 (35.6) | 21 (46.7) |  |
| Years of employment |  |  |  |
| <3 | 62 (27.6) | 9 (20.0) | 0.559 |
| 3-9 | 87 (38.7) | 20 (44.4) |  |
| ≥10 | 76 (33.8) | 16 (35.6) |  |
| Weekly hours of work |  |  |  |
| ≤40 | 74 (32.9) | 19 (42.2) | 0.229 |
| >40 | 151 (67.1) | 26 (57.8) |  |
| Job burnout |  |  |  |
| No burnout | 72 (32.0) | 10 (22.2) | 0.424 |
| Mild to moderate burnout | 103 (45.8) | 24 (53.3) |  |
| Severe burnout | 50 (22.2) | 11 (24.4) |  |
| Significant depression symptoms |  |  |  |
| No | 86 (38.2) | 11 (24.4) | 0.079 |
| Yes | 139 (61.8) | 34 (75.6) |  |
| Significant anxiety symptoms |  |  |  |
| No | 118 (52.4) | 21 (46.7) | 0.479 |
| Yes | 107 (47.6) | 24 (53.3) |  |
| Insomnia |  |  |  |
| None or subclinical insomnia | 179 (79.6) | 38 (84.4) |  |
| Moderate to severe insomnia | 46 (20.4) | 7 (15.6) |  |
| Stress Mean (S.D.) | 21.27 (4.49) | 21.36 (4.16) | 0.907 |
| Depression Mean (S.D.) | 11.34 (4.37) | 12.20 (4.68) | 0.233 |
| Anxiety Mean (S.D.) | 9.44 (3.97) | 10.04 (4.21) | 0.357 |
| Insomnia Mean (S.D.) | 10.32 (5.49) | 10.00 (5.38) | 0.721 |
| Burnout Mean (S.D.) | 3.30 (1.37) | 3.49 (1.29) | 0.401 |
| Positive effect Mean (S.D.) | 25.56 (5.68) | 24.76 (5.44) | 0.380 |
| Self-kindness Mean (S.D.) | 16.97 (3.78) | 16.29 (4.03) | 0.277 |

*The chi-square test was used for categorical variables and the t-test was used for continuous variables.

**Table S4. Trend test of changes in the scores of primary and secondary outcomes at T2 from T0 among the intervention group (N = 117)**

| Variable | Rarely practice | Occasionally practice | Sometimes practice | Often practice | *P* for trend |
| --- | --- | --- | --- | --- | --- |
| Changes in stress score | -4.32 (7.59) | -4.73 (5.54) | -4.96 (5.96) | -8.38 (7.46) | 0.049 |
| Changes in self-kindness score | 2.84 (3.38) | 1.79 (2.76) | 0.72 (2.79) | 2.57 (3.54) | 0.528 |
| Changes in positive affect score | 2.89 (4.39) | 3.75 (4.52) | 3.44 (5.47) | 4.48 (8.02) | 0.411 |

**Table S5. Trend Test of Changes in Scores of Primary and Secondary Outcomes at T3 from T0 in the Intervention Group (N = 110)**

| Variable | Rarely practice | Occasionally practice | Sometimes practice | Often practice | *P* for trend |
| --- | --- | --- | --- | --- | --- |
| Changes in stress score | -3.69 (7.27) | -5.30 (6.05) | -8.17 (6.88) | -12.60 (11.71) | 0.002 |
| Changes in depression score | -1.06 (5.25) | -2.49(4.63) | -5.47(5.08) | -7.20(6.91) | <0.001 |
| Changes in anxiety score | -0.31(5.15) | -2.34 (4.37) | -4.17 (3.79) | -6.60 (4.45) | <0.001 |
| Changes in insomnia score | -1.50 (4.12) | -2.58 (4.30) | -3.14 (5.46) | -6.80 (7.56) | 0.061 |
| Change in burnout score | -0.31 (1.01) | -0.17 (1.54) | -0.58 (1.56) | -1.60 (2.07) | 0.095 |
| Changes in self-kindness score | 1.31 (3.42) | 1.85 (3.10) | 2.47 (3.90) | 3.00 (2.55) | 0.172 |
| Changes in positive affect score | 2.56 (4.90) | 3.00 (5.66) | 4.22 (4.42) | 10.00 (9.72) | 0.021 |

**Table S6. Intervention Group Completion Effect Evaluation at T2 (N = 117)**

| Item | N (%) |
| --- | --- |
| What is the status of your practice with each chapter of our intervention materials since the intervention period to date? |  |
| Rarely practice (0-5 times/month) | 19 (14.5) |
| Occasionally practice (5-10 time/month) | 52 (48.2) |
| Sometimes practice (11-15 times/month) | 25 (32.7) |
| Practice often (16 times and more/month) | 21 (4.5) |

**Table S7.** **Generalised Estimating Equations Predict Stress at Different Time Points and Other Secondary Outcomes using data with multiple imputation.**

| Variable | Model 1 | | Model 2 | | Model 3 | |
| --- | --- | --- | --- | --- | --- | --- |
|  | β (95%CI) | *p* | β (95%CI) | *p* | β (95%CI) | *p* |
| Primary outcome |  |  |  |  |  |  |
| **Stress** |  |  |  |  |  |  |
| Group |  |  |  |  |  |  |
| Control | Ref | 0.025 | Ref | 0.022 | Ref | 0.660 |
| Intervention | -1.33 (-2.50, -0.17) |  | -1.29 (-2.39, -0.19) |  | 0.42 (-1.45, 2.30) |  |
| Time | -0.48 (-0.86, -0.11) | 0.014 | -0.48 (-0.86, -0.11) | 0.014 | -0.05 (-0.65, 0.54) | 0.860 |
| Baseline stress | Na | Na | 0.29 (0.13, 0.45) | <0.001 | 0.29 (0.13, 0.45) | <0.001 |
| Group*Time | Na | Na | Na | Na | -0.86 (-1.69, -0.02) | 0.057 |
| Secondary outcome |  |  |  |  |  |  |
| **Self-kindness** |  |  |  |  |  |  |
| Group |  |  |  |  |  |  |
| Control | Ref | 0.028 | Ref |  | Ref |  |
| Intervention | 0.73 (0.09, 1.37) |  | 0.73 (0.18, 1.28) | 0.011 | 0.54 (-0.92, 2.00) | 0.479 |
| Time | 0.12 (-0.13, 0.38) | 0.335 | 0.12 (-0.13, 0.38) | 0.335 | 0.08 (-0.34, 0.49) | 0.718 |
| Baseline self-kindness | Na | Na | 0.37 (0.28, 0.47) | <0.001 | 0.37 (0.28, 0.47) | <0.001 |
| Group*Time | Na | Na | Na | Na | 0.10 (-0.58, 0.77) | 0.785 |
| **Positive affect** |  |  |  |  |  |  |
| Group |  |  |  |  |  |  |
| Control | Ref | 0.055 | Ref |  | Ref |  |
| Intervention | 1.06 (-0.02, 2.14) |  | 1.26 (0.28, 2.23) | 0.013 | 0.29 (-1.37, 1.95) | 0.733 |
| Time | 0.04 (-0.55, 0.63) | 0.908 | 0.04 (-0.55, 0.63) | 0.908 | -0.21 (-0.92, 0.51) | 0.583 |
| Baseline positive affect | Na | Na | 0.37 (0.26, 0.48) | <0.001 | 0.37 (0.26, 0.48) | <0.001 |
| Group*Time | Na | Na | Na | Na | 0.48 (-0.29, 1.25) | 0.221 |
